# Phylogeography and demographic history of the Chagas disease vector *Rhodnius nasutus* (Hemiptera: Reduviidae) in the Brazilian Caatinga biome

**DOI:** 10.1371/journal.pntd.0006731

**Published:** 2018-09-24

**Authors:** Tatiana Peretolchina, Márcio G. Pavan, Jessica Corrêa-Antônio, Rodrigo Gurgel-Gonçalves, Marli M. Lima, Fernando A. Monteiro

**Affiliations:** 1 Laboratório de Epidemiologia e Sistemática Molecular, Instituto Oswaldo Cruz, Fiocruz, Rio de Janeiro, Brazil; 2 Laboratory of Molecular Systematics, Limnological Institute of the Siberian Branch of the Russian Academy of Sciences, Irkutsk, Russia; 3 Laboratório de Mosquitos Transmissores de Hematozoários, Instituto Oswaldo Cruz, Fiocruz, Rio de Janeiro, Brazil; 4 Laboratório de Parasitologia Médica e Biologia de Vetores, Faculdade de Medicina, Universidade de Brasília, Brasília, Distrito Federal, Brazil; 5 Laboratório de Ecoepidemiologia da doença de Chagas, Instituto Oswaldo Cruz, Fiocruz, Rio de Janeiro, RJ, Brazil; Universidad de Buenos Aires, ARGENTINA

## Abstract

**Background:**

*Rhodnius nasutus*, a vector of the etiological agent *Trypanosoma cruzi*, is one of the epidemiologically most relevant triatomine species of the Brazilian Caatinga, where it often colonizes rural peridomestic structures such as chicken coops and occasionally invades houses. Historical colonization and determination of its genetic diversity and population structure may provide new information towards the improvement of vector control in the region. In this paper we present thoughtful analyses considering the phylogeography and demographic history of *R*. *nasutus* in the Caatinga.

**Methodology/Principal findings:**

A total of 157 *R*. *nasutus* specimens were collected from *Copernicia prunifera* palm trees in eight geographic localities within the Brazilian Caatinga biome, sequenced for 595-bp fragment of the mitochondrial cytochrome b gene (cyt b) and genotyped for eight microsatellite loci. Sixteen haplotypes were detected in the cyt b sequences, two of which were shared among different localities. Molecular diversity indices exhibited low diversity levels and a haplotype network revealed low divergence among *R*. *nasutus* sequences, with two central haplotypes shared by five of the eight populations analyzed. The demographic model that better represented *R*. *nasutus* population dynamics was the exponential growth model. Results of the microsatellite data analyses indicated that the entire population is comprised of four highly differentiated groups, with no obvious contemporary geographic barriers that could explain the population substructure detected. A complex pattern of migration was observed, in which a western Caatinga population seems to be the source of emigrants to the eastern populations.

**Conclusions/Significance:**

*R*. *nasutus* that inhabit *C*. *prunifera* palms do not comprise a species complex. The species went through a population expansion at 12–10 ka, during the Holocene, which coincides with end of the largest dry season in South America. It colonized the Caatinga in a process that occurred from west to east in the region. *R*. *nasutus* is presently facing an important ecological impact caused by the continuous deforestation of *C*. *prunifera* palms in northeast Brazil. We hypothesize that this ecological disturbance might contribute to an increase in the events of invasion and colonization of human habitations.

## Author summary

Chagas disease is endemic to Latin America and the Caribbean and it is estimated that 6–7 million people are infected with the etiological agent *Trypanosoma cruzi*. Although new community-based ecosystem management (ecohealth) initiatives have been implemented, vector control based on insecticide-spraying of households remains one of the most effective strategies to diminish parasite transmission to humans. However, this strategy is not sustainable where native triatomine species are capable of colonizing peridomestic structures and invading human dwellings. The application of molecular markers with the potential of recovering both historical and contemporary information on vector population structure and diversity can improve the understanding of vector dissemination and thus contribute to the development of better disease control strategies. In this study we analyzed *Rhodnius nasutus* populations endemic to the Brazilian Caatinga biome using two sets of markers: a fragment of the mitochondrial gene cytochrome b and eight nuclear microsatellite loci. The information generated and described herein is important as it may contribute to the advancement of our understanding of Chagas disease vector ecology and phylogeography.

## Introduction

Chagas disease is caused by the protozoan *Trypanosoma cruzi* (Kinetoplastida, Trypanosomatidae) and transmitted primarily through the feces of infected triatomine bugs (Hemiptera, Reduviidae) [1]. Endemic to Latin America and the Caribbean, it is estimated that approximately 6–7 million people are infected worldwide [2].

Although curable when treated early with antiparasitics in the acute phase, there is no vaccine available and treatment of chronic patients only reduces serum parasite detection, but not cardiac clinical complications [3]. Therefore, control programs have placed their efforts on the elimination of domestic vectors, by spraying insecticides indoors [4]. A recurrent problem seems to be the constant re-infestation of insecticide-treated households by abundant native triatomine species, which consists of a major challenge for the consolidation of Chagas disease control efforts [5, 6]. This dreadful scenario is often reported in Brazil where native *T*. *cruzi*-infected triatomines continuously invade houses in both rural and urban areas maintaining the risk of transmission [7, 8].

The Caatinga and the Cerrado biomes together, harbor most of the triatomine species diversity in Brazil [9]. Of the 68 triatomine species known to occur in the country, 10 have successfully adapted to the harsh droughts imposed by the Caatinga. *Rhodnius nasutus* Stål, 1859 is one of these species, frequently reported in bird and mammal nests on the crown of *Copernicia prunifera* (Carnaúba) palm trees in the vicinity of human habitations. It is often naturally infected with the *T*. *cruzi* parasite and is capable of invading rural and urban houses and colonizing peridomestic structures [10, 11]. It can also occur in *Attalea speciosa*, *Mauritia flexuosa*, *Syagrus oleracea* and *Acrocomia intumescens* palm trees [12, 13]. Interestingly, *R*. *nasutus* has also been reported to occur in *Licania rigida* (Oiticica) dicotyledon trees typical of northeast Brazil [10, 14].

Determination of *R*. *nasutus* population structure in the Caatinga biome and estimation of present gene flow may contribute to a better understanding of the species' historical colonization process as well as provide new information towards the improvement of vector control in the region. In this paper we present the phylogeography and demographic history of this Chagas disease vector in the Caatinga inferred with mtDNA (cyt b) and nuclear (microsatellites) markers.

## Materials and methods

### Insect collection

Two-hundred and twenty-eight *Rhodnius* specimens were collected after the inspection of 53 *Copernicia prunifera* palms in eight geographic localities within the Brazilian Caatinga biome (Table 1). The two closest collection sites are located 40 km apart (Altos and Campo Maior), while the most distant are 670 km apart (Parnaíba and Serra Talhada). The Caatinga biome covers nearly 85,000 km^2^ of the northeast region, belongs to the seasonally dry tropical forests phytogeographic unit [15], and is a species-rich xeric environment [16, 17].

**Table 1:** Sampling localities, number of *Rhodnius*-infested *Copernicia prunifera* palm trees and sample sizes of *R*. *nasutus* used in each molecular analysis.

Sampling locations	Geographical coordinates	Palms			Triatomines			Year	Code
		N_P_	Infestation (%)	Habitat^1^	N_R_	N*	N^┼^		
1. Altos, Piauí	05°02´S 42°28´W	5	80	Sylvatic	12	4	2	2010	ALT
2. Campo Maior, Piauí	04°49´S 42°10´W	8	50	Sylvatic	21	16	20	2010	CAM
3. Parnaíba, Piauí	02°54´S 41°46´W	5	80	Peridomestic	26	18	18	2010	PAR
4. Piracuruca, Piauí	03°55´S 41°43´W	4	75	Peridomestic	9	9	8	2010	PIR
5. Jaguaruana, Ceará	04°50´S 37°46´W	5	-	Peridomestic	44	32	44	2004	JAG
6. Serra Talhada, Pernambuco	07°59´S 38°17´W	7	57	Sylvatic	23	15	13	2010	STA
7. Sousa, Paraíba	06°45´S 38°13´W	6	100	Peridomestic	44	28	26	2010	SOU
8. Carnaúba dos Dantas, Rio Grande do Norte	06°33´S 36°35´W	13	54	Sylvatic	49	25	24	2010	CAR
	**TOTAL**	**53**			**228**	**147**	**155**		

N_P_: number of palms infested with *Rhodnius* specimens

^1^: Sylvatic–forested areas >200m-distant from human dwellings, Peridomestic–deforested area <200m-distant from human dwellings; N_R_: number of *Rhodnius* specimens collected; N*: sample sizes used in microsatellite analyses; N^┼^: sample sizes used in cyt b analyses.

Insect field captures were approved by the *Instituto Chico Mendes de Conservação da Biodiversidade* (ICMBio) and were carried out following Gurgel-Gonçalves et al. [18]. Briefly, after reaching up to the palm tree crowns with a ladder, leaves and fibers were pruned and placed into plastic bags. The collected material was then lowered to the ground and scattered over a piece of white cloth to facilitate bug detection. *Rhodnius nasutus* specimens were identified morphologically based on Lent and Wygodzinsky [19] and confirmed by molecular taxonomy based on a 682 base pair (bp) fragment of the mitochondrial cytochrome b gene (cyt b) [20]. Sequences generated with the amplification of this fragment were also used in the population genetics and phylogeographic analyses described in the next section.

### Mitochondrial cyt b analyses

DNA extraction was performed using the Promega Wizard Genomic DNA extraction kit (Promega, Madison, Wisconsin, USA) following the manufacturers recommendations. A 682-bp fragment of the cyt b gene was PCR-amplified as described in Monteiro *et al*. [20]. Amplicons were purified with the Hi Yield Gel/PCR DNA extraction kit (Real Biotech, Banqiao, Taipei, Taiwan), or following the purification protocol with PEG-NaCl 20% - 5mM (modified from [21]). Both DNA strands were subjected to Sanger sequencing reactions with the ABI Prism BigDye Terminator v3.1 Cycle Sequencing kit (Thermo Fisher Scientific, Walthan, Massachusetts, USA) and run on an ABI 3730 automated sequencer (Applied Biosystems, Foster City, California, USA). The removal of primer sequences, editing of both forward and reverse strands, and the generation of a consensus sequence for each sample were performed with Seqman Lasergene v. 7.0 (DNAStar, Inc., Madison, Wisconsin, USA). Consensus sequences were aligned to published ortholog sequences from other members of the ‘robustus lineage’ (*Rhodnius robustus* I-V, *R*. *prolixus*, *R*. *neglectus*, *R*. *nasutus* and *R*. *barretti*) and analyzed to confirm species identity based on genetic distances using Mega v. 5 [22]. Taxonomic identification was achieved based on the genetic similarity with other *Rhodnius* sequences available in the GenBank public database through the Basic Local Alignment Search Tool (https://blast.ncbi.nlm.nih.gov/Blast.cgi), considering the following parameters: percentage of identity and coverage, e-value, and also Bayesian phylogenetic analyses.

A Bayesian phylogenetic cyt b tree based on the 147 samples of morphologically-identified *R*. *nasutus* and the other members of the ‘robustus lineage’ was inferred in BEAST v. 1.8 [23]. *Rhodnius barretti* [24] was used to root the maximum clade credibility tree. The best fit model of substitution was determined using jModeltest v. 2 [25]. We tested whether a strict or a relaxed molecular clock best fitted our data through a Bayesian random local clock analysis (RLC) [26]. Three independent runs were performed for 10^9^ generations, sampling every 20,000 generations. Convergence of parameters and proper mixing were confirmed through the calculation of effective sample sizes (ESS) in Tracer v. 1.6 [27], and ESS estimates above 10^4^ were considered appropriate [28].

Gene tree and species tree were reconstructed with *BEAST [29]. Information in the literature about the taxonomic identification of DNA sequences retrieved from GenBank was used to assign prior information on each sequence to a particular species. The 147 cyt b sequences from field-collected specimens without molecular identification were labeled as “*Rhodnius* sp.”. Suggested divergence rate for triatomines of 1.1 to 1.8% per Myr was used as a prior with normal distribution [30].

Molecular indices of haplotype diversity (Hd) and nucleotide diversity (π) were computed in DnaSP v. 5 [31] for each population and sequence divergence between populations were calculated in Mega v. 5 [22]. A median-joining network [32] was constructed with Network v. 4.6 (Fluxus Technology Ltd. 2008) for a better visualization of the relationships between *R*. *nasutus* cyt b haplotypes. Since samples from Jaguaruana were collected in 2004 and all other samples in 2010, we tested whether this could have an impact in our study by carrying out an analysis of variance (AMOVA) grouping samples according to the collection date with Arlequin v. 3.5 [33].

Levels of genetic differentiation among populations were determined with Wright’s pairwise *F*_ST_ comparisons [34] in Arlequin v. 3.5 [33]. Correction for multiple testing was done with the false discovery rate (FDR) method [35] under 5% of significance level (P-value = 0.013). The coefficient of determination R^2^ to evaluate the fit of data to theoretical models of distribution of genetic distances under different demographic scenarios was calculated in the R environment [36]. Deviations from neutrality were assessed with Fu’s Fs [37] and Tajima’s D [38] tests in DnaSP v. 5 [31]. Significant and negative values of both tests indicate population size expansion or purifying selection. Mismatch distribution tests were also used to infer possible demographic and spatial expansions [39].

We also used Beast v. 2.4 [40] to reconstruct a Bayesian skyline plot (BSP) and test which of the two different demographic models (constant population size or exponential growth) better explained the demographic history of *R*. *nasutus*. The best model was selected through the comparison of marginal likelihood, calculated with the path sampling algorithm [41] under 10^6^ MCMC chain length and 60 steps, based on Bayes Factors (BF) [42]. In all cases, results from two independent runs (2 x 10^7^ generations with the first 2 x 10^6^ discarded as burn-in and parameter values sampled every 2 x 10^3^ generations) were analyzed with Tracer v. 1.6 [27]. Convergence of parameters and proper mixing were confirmed through the calculation of ESS, and estimates above 10^4^ were considered appropriate [28]. Because only a single species was analyzed (and thus it is highly probable that specimens from all populations evolve at a single rate), we imposed a strict clock in the analysis. Suggested divergence rates for triatomines of 1.1 to 1.8% per Myr were used [30]. We matched the *R*. *nasutus* demographic history to paleoclimatic events estimated through oxygen isotope marine paleotemperature records [43], used as etalon for the study of global climate changes.

### Microsatellite analyses

Microsatellite alleles of eight loci were amplified in a total reaction volume of 10 uL containing 1 unit of *TaqGold* DNA polymerase, 2 mM of each dNTP, 1.5 mM MgCl_2_, 10 mM of magnesium free Buffer 10X (Thermo Fisher Scientific Co., Walthan, Massachusetts, USA), 5 pmol of each primer and approximately 10 ng of extracted DNA. PCR conditions were as follows: 1 min at 95°C, 30 cycles of 30 s at 94°C, 30 s at Ta °C and 45 s at 72°C, and 72°C for 30 min; Ta is the annealing temperature for each locus (S1 Table).

All PCR products were run with a size standard GS500 LIZ on an ABI 3730xl Genetic Analyzer, and allele fragment lengths quantified using the Peak Scanner software v. 1.0 (Applied Biosystems, Foster City, California, USA). Because these primers are heterologous (designed to amplify microsatellite loci of other *Rhodnius* species) [44–46], the orthology of microsatellite regions and repetition motifs were molecularly validated. Amplified loci were submitted to Sanger sequencing reactions with the ABI Prism BigDye Terminator v3.1 Cycle Sequencing kit (Thermo Fisher Scientific, Walthan, Massachusetts, USA) and run on an ABI 3730 automated sequencer (Applied Biosystems, Foster City, California, USA).

Microsatellite genotypes were screened for likely scoring errors, allele dropout, and presence of null alleles with Micro-Checker v. 2.2 [47]. Number of shared genotypes, number of private alleles for each sampling site and Shannon’s allele information index (^S^*H*_A_) as a measure of gene diversity were performed with GenAlEx v. 6.5 [48]. Deviations from Hardy–Weinberg equilibrium and tests of linkage disequilibrium for each locus were performed with Arlequin v. 3.5 software [33].

Genetic differentiation between populations (pairwise *F*_ST_) between sampling localities and inbreeding coefficients (*F*_IS_) were carried out with Arlequin v. 3.5 software [33]. Sampling localities for which *F*_ST_ was non-significant were considered as belonging to the same population. Mutual index (^S^*H*_UA_) [49] were also estimated with GenAlEx v. 6.5 [48].

The Bayesian clustering program Structure v. 2.3 [43] was used to estimate population assignment without prior assumptions of population subdivision. We used the *admixture model* due to the lack of information about the ancestry of the field-collected specimens, with correlated allele frequencies, which means that these frequencies in different populations are likely to be similar as a consequence of migration or shared ancestry [50]. Burn-in and simulation were set at 2.5 x 10^5^ iterations and 10^6^ Markov Chain Monte Carlo (MCMC) generations, respectively. Ten independent runs were performed for each value of K (for 2–8), as suggested by Pritchard et al. [50]. The most likely value of K was estimated with the ΔK method [51].

The Ewens–Watterson neutrality test [52], which relies on the comparison of observed and expected homozygosity, was performed with the Arlequin v. 3.5 software [33].The program Bottleneck v. 1.2 [53] was used to test bottleneck events by evaluating deviations of mutation-drift equilibrium. Three different mutation models of microsatellite evolution were employed: Infinite Allele model (IAM), Stepwise Mutation Model (SMM), and Two-Phased model (TPM), which is an intermediate to the SMM and IAM as it incorporates the mutational process of the former, but allows for mutations of a larger magnitude to occur. The significance was assessed with the Wilcoxon sign-rank test [54], a more powerful and robust test when few polymorphic loci are available (< 20).

We also constructed a population network with EDENetwork v. 2.1 [55] to determine possible source and sink populations of *R*. *nasutus*. Basically, this analysis reconstructs a minimum-spanning tree based on the pairwise *F*_ST_ matrix and calculates three different parameters: (a) degree, which is defined as the number of connections a node has in the network, summarizing which populations are exchanging migrants; (b) clustering, which measures the number of subpopulations within a population; and (c) betweenness, which is the number of shortest paths between populations, reflecting areas of intense gene flow. These measures allow identifying “source” and “sink” populations (i.e. those with the highest degree and betweenness) [56].

To investigate the patterns of gene flow between the populations defined by Structure we performed a Bayesian approach implemented in Migrate-n v. 3.2 [57]. Parameters were first estimated under a full migration model that allowed gene flow to occur among all regions. Then we tested 10 reduced models (based on *F*_ST_ results) representing different patterns of migration. Migrate-n analysis was performed with a single long cold chain and four hot swapping chains using a static heating scheme (temperature: 1.0, 1.5, 3.0, 10^6^), with two independent runs, sampling at every 100^th^ step for a total of 2 x 10^5^ recorded steps (burn-in = 3 x 10^4^ steps). The 10 migration models were compared based on their marginal likelihood and probability using thermodynamic integration with Bezier approximation (implemented in Migrate-n) according to Beerli and Palczewski [58]. The plot for visualizing of migration pattern inferred by Migrate-n analysis was drawn in the R environment [36] using Migest Package [59]. The migration scenario with highest probability was tested through an AMOVA with Arlequin v. 3.5 [33].

Isolation by distance was estimated for cyt b and microsatellite data through linear regression analyses of dependence, comparing the logarithms of genetic distances (pairwise *F*_ST_ values) [60], and geographic distances between the eight localities, in the R environment [36]. The significance level of hypothesis testing was set at α = 0.05.

## Results

### Morphological taxonomy and ecology

Thirty-three of the 53 *C*. *prunifera* palm trees investigated (62%) were infested with *Rhodnius* specimens. Higher infestation percentages were detected in deforested areas (Table 1). Thirty-three palm trees were sampled in four forested locations and showed lower infestation percentages (Mean: 58%, range = 50–80%) than the 15 palm trees collected in four deforested areas (Mean: 87%, 75–100%). From the 228 *Rhodnius* specimens collected, 163 insects were morphologically identified as *Rhodnius nasutus*.

### Mitochondrial cyt b analyses

Only two of the DNAs extracted from all 157 specimens resulted in unsuccessful amplification of the desired cyt b fragment. Eight specimens exhibited low-quality sequences (i.e. < 500-bp) and thus were discarded from the analysis. Therefore, our dataset included 147 cyt b sequences of 595-bp long from *R*. *nasutus* individuals sampled from eight localities in the Brazilian Caatinga (Table 1).

Pairwise comparison of the sequences obtained with those available in GenBank revealed 99–100% identity with another *R*. *nasutus* “reference” sequence (JX273155) generated by our group [24]. This sequence was obtained from a specimen collected in Jaguaruana, Ceará, where the morphologically similar species *R*. *neglectus* is not known to occur [61].

**Fig 1:**
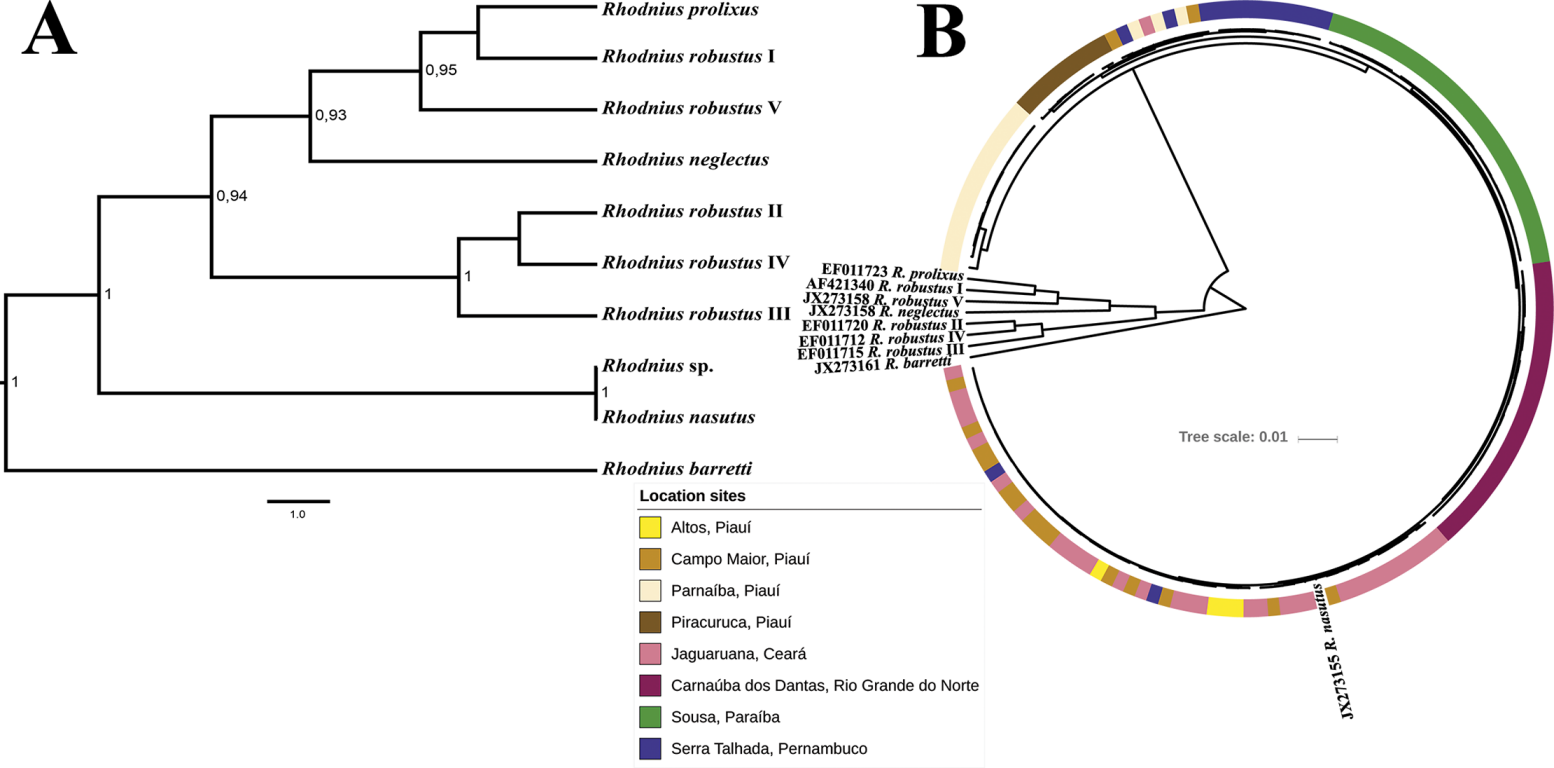
A–Maximum clade credibility species tree reconstructed with 595-bp cyt b sequences of 156 *Rhodnius* specimens. Posterior probabilities above 0.95 are show for key nodes. B–Coalescent gene tree with 147 sequences of 595-bp cyt b. GenBank accession numbers of *R*. *nasutus* sequenced in this study are MG734978-MG735124. The accession numbers of the sequences retrieved from GenBank are shown on the tips of their respective tree stems.

Hasegawa-Kishino-Yano (HKY [62]) was selected as the best evolutionary model for the data, following the Akaike and Bayesian Information criteria. RLC analysis showed that a “strict” clock is more suitable to our cyt b dataset (rate change: median = 0; variance = 0.67; ESS = 4020). Bayesian species tree (Fig 1) revealed that all sequences from *Rhodnius* specimens collected formed a monophyletic and well-supported clade with the *R*. *nasutus* reference sequence (PP = 1.0), corroborating further their taxonomic identity. A bayesian coalescent gene tree disclosed the short branches (i.e. low sequence divergence) among *R*. *nasutus* samples. Although posterior probabilities for *R*. *nasutus* clades were low (PP < 0.9), it is noticeable that only the *R*. *nasutus* sequences from Carnaúba dos Dantas and Sousa clustered in separate clades without the presence of at least one sequence from another locality. Sequences from the other six localities clustered together in other three different clades.

**Fig 2:**
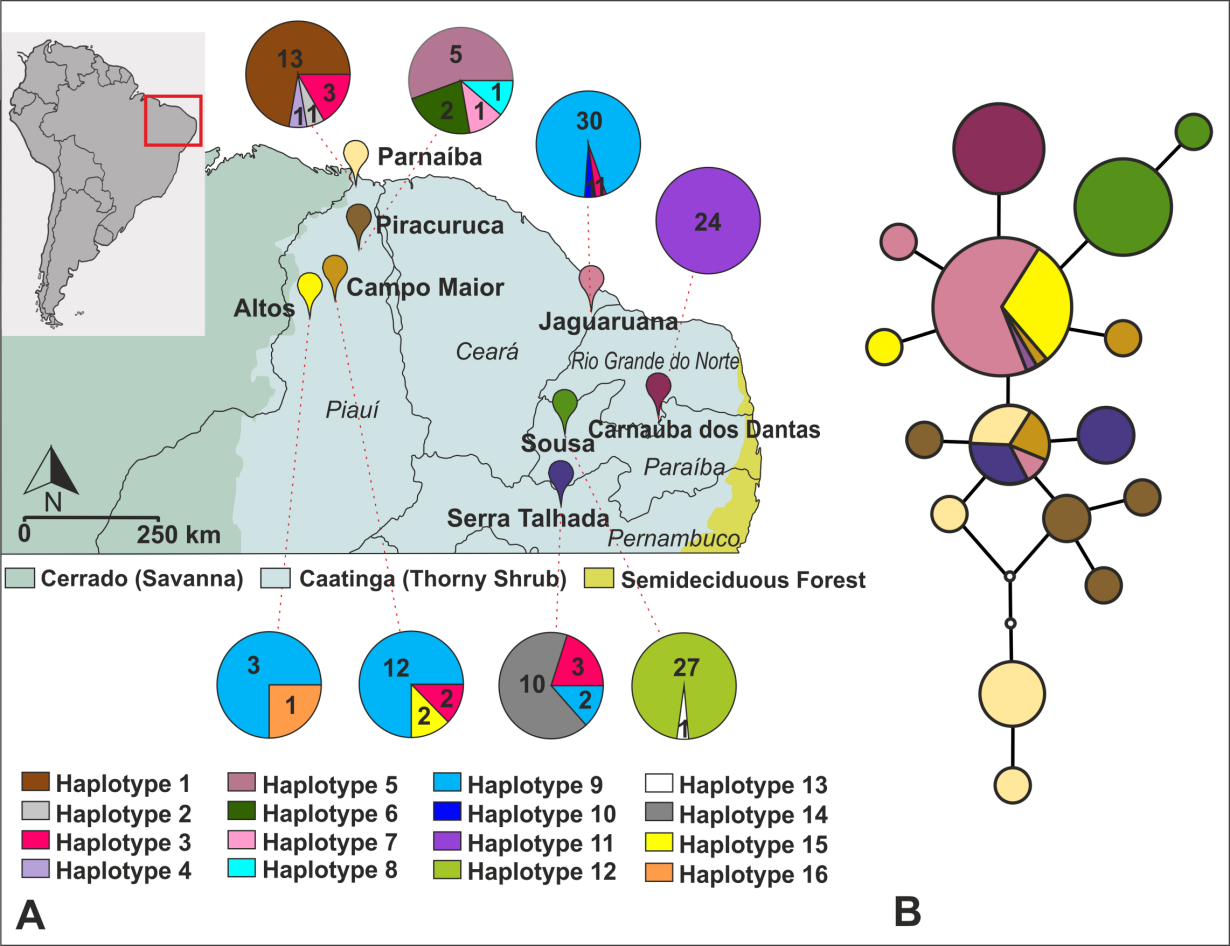
A–Geographical distribution of *Rhodnius nasutus* cytb haplotypes in each sampled locality in northeastern Brazil. Slice sizes are proportional to haplotype frequency. B—Haplotype network based on cyt b sequences. Circle sizes are proportional to haplotype frequency.

AMOVA analysis between Jaguaruana and the other populations did not indicate any sampling effect of collecting at different times as genetic differences between these groups were not significant (Φ_ST_ = 0.18, P = 0.98). Therefore, these samples were included in further analyses. Molecular divergence of *R*. *nasutus* cyt b sequences varied between 0%-0.8% (S2 Table), as expected for intraspecific comparisons in *Rhodnius* species [20]. Pairwise comparisons within and between localities ranged from 0% to 0.2% and 0.2% to 0.8%, respectively. Overall, the most divergent sequences were present in Parnaíba (mean divergence = 0.62%) and the less divergent sequences were in Jaguaruana (mean divergence = 0.27%). Inspection of the sequences revealed 15 polymorphic sites and 16 haplotypes, with two haplotypes (3 and 9) shared between different localities (Fig 2A). Molecular diversity indices (Table 2) showed high haplotype diversity (Hd = 0.45–0.83 ± 0.10), but low nucleotide diversity between haplotypes (π = 0.0032 ± 0.0002). The highest haplotype diversities were found in Piracuruca (N = 9, Hd = 0.694±0.147) and Altos (N = 4, Hd = 0.667±0.204), and the lowest haplotype diversity in Sousa (N = 28, Hd = 0.071±0.065). All sequences from Carnaúba dos Dantas (N = 25) were identical. The highest nucleotide diversities were found in sequences from Parnaíba (N = 18, π = 0.00253±0.00073) and Piracuruca (N = 9, π = 0.00205±0.00058), and the lowest nucleotide diversities in sequences from Sousa (N = 28, π = 0.00012±0.00011) and Jaguaruana (N = 32, π = 0.00021±0.00013). Pairwise population *F*_ST_ estimates are given in Table 3. Most of the comparisons revealed high and significant values (> 0.6). Non-significant *F*_ST_ values were observed only when the populations of Altos, Campo Maior and Jaguaruana were compared. The excessive number of high *F*_ST_ values is due to the presence of 14 (out of 16) unique haplotypes.

**Table 2:** Molecular diversity indices for the *R*. *nasutus* sequences collected in eight localities.

Localities	N	S	π (±SD)	h	Hd (± SD)
**1.** ALT	4	1	0.00112 (± 0.00034)	2	0.667 (± 0.204)
**2.** CAM	16	2	0.00078 (± 0.00028)	3	0.433 (± 0.138)
**3.** CAR	25	0	0.00000	1	0.000
**4.** JAG	32	2	0.00021 (± 0.00013)	3	0.123 (± 0.078)
**5.** PAR	18	5	0.00253 (± 0.00074)	4	0.471 (± 0.130)
**6.** PIR	9	4	0.00205 (± 0.00058)	4	0.694 (± 0.147)
**7.** SOU	28	1	0.00012 (± 0.00011)	2	0.071 (± 0.065)
**8.** STA	15	2	0.00124 (± 0.00033)	3	0.553 (± 0.126)
***R*. *nasutus***	147	15	0.00322 (± 0.00023)	16	0.826 (± 0.018)

N = sample size, S = number of polymorphic sites, π = nucleotide diversity, SD = standard deviation, h = number of haplotypes; Hd = haplotype diversity.

**Table 3:** Pairwise *F*_ST_ values for microsatellite data (above the diagonal) and cyt b data (below the diagonal).

Localities	1	2	3	4	5	6	7	8
**1**-ALT	**-**	0.07	0.24*	0.11	0.30*	0.49*	0.25*	0.64*
**2**-CAM	0.20	-	0.08	0.04	0.27*	0.32*	0.26*	0.53*
**3**-PAR	0.74*	0.77*	-	0.16	0.42*	0.30*	0.36*	0.64*
**4**-PIR	0.62*	0.66*	0.58*	-	0.28*	0.40*	0.22*	0.55*
**5**-CAR	0.93*	0.85*	0.88*	0.89*	-	0.51*	0.31*	0.19
**6**-SOU	0.89*	0.83*	0.88*	0.89*	0.98*	-	0.47*	0.65*
**7**-STA	0.71*	0.69*	0.74*	0.58*	0.92*	0.91*	-	0.53*
**8-**JAG	0.44	0.06	0.85*	0.82*	0.93*	0.91*	0.83*	-

* Significant results (P < 0.013)

Haplotypes derived from cyt b sequences revealed a network (Fig 2B) with two central haplotypes (haplotypes 3 and 9) that are very abundant and widespread, and to which several less common haplotypes are closely related (1–2 mutational steps). The most frequent haplotype (Haplotype 9, N = 46) is shared with specimens from Altos, Campo Maior and Jaguaruana, and the other haplotype (Haplotype 3, N = 9) is shared with specimens from Campo Maior, Jaguaruana, Parnaíba and Serra Talhada. This type of haplotype connection suggests population expansion or retention of ancestral polymorphism with little migration, evidenced by the geographically restricted haplotypes.

**Fig 3:**
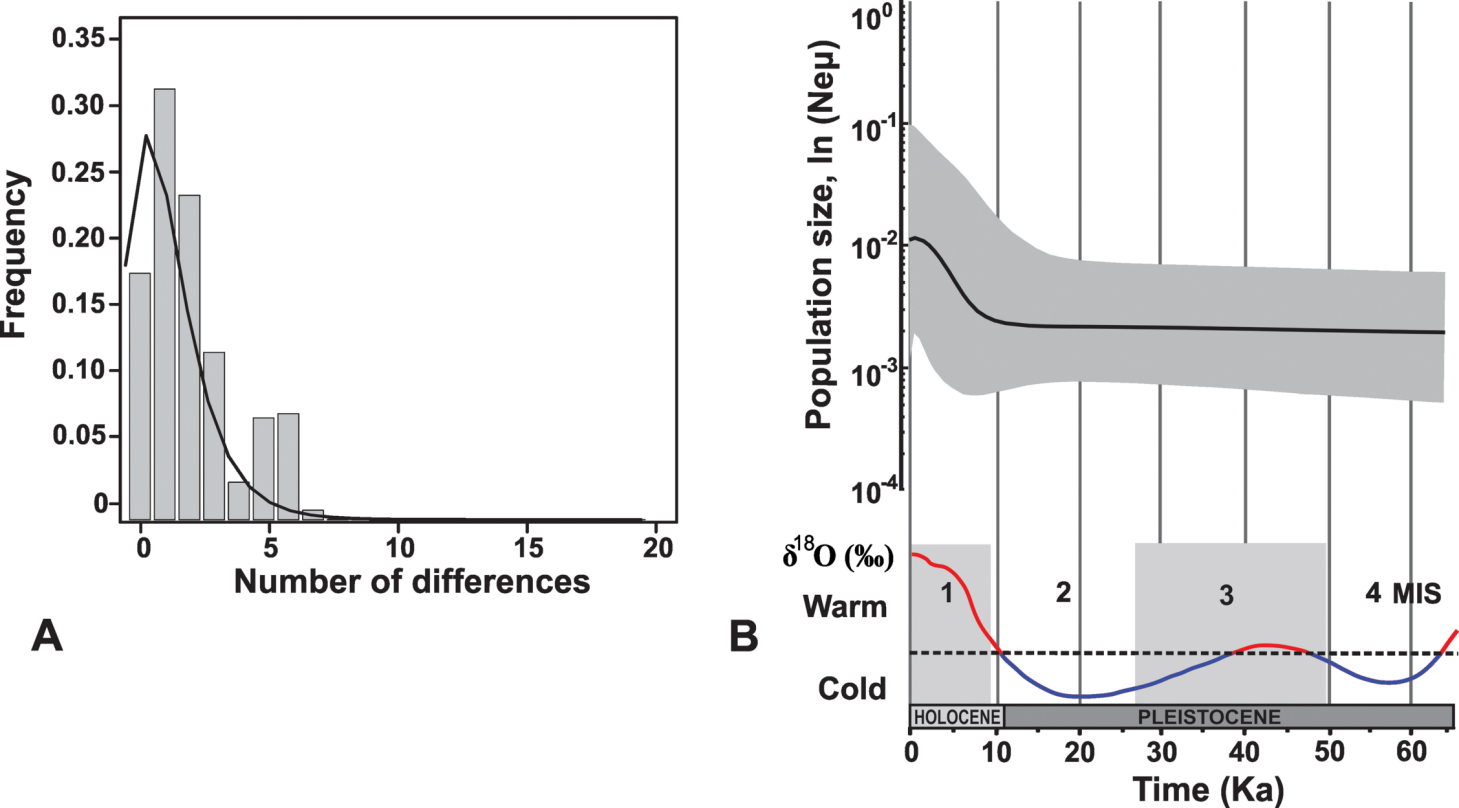
A—Mismatch distribution for *R*. *nasutus* based on cyt b sequences. Bars represent observed values, lines represent expected values under the model of sudden population growth; B—Calibrated demographic history of *R*. *nasutus* and reconstructed paleoclimatic history. Shaded areas correspond to 1st and 3rd Marine Isotope Stages.

Considering that all *R*. *nasutus* sequences are separated by small genetic distances (most haplotypes are connected by a single mutational step), neutrality tests, mismatch distribution and BSP analyses were performed with the entire dataset. Mismatch distribution analysis (Fig 3A) exhibits a relatively good fit to the expected mismatch distributions under the model of population expansion (coefficient of determination R^2^ for population growth model is 0.95 (p = 0.008) while for constant population size model is 0.72 (p = 0.160)). Neutrality tests did not indicate significant departures from neutrality (Fu’s *Fs* = -4.27, *p* = 0.085; Tajima’s *D* = -0.75, *p* = 0.246). Bayesian analyses of population growth revealed that marginal likelihood for constant population was -1240.82 and for exponential growth was -1228.27. Thus, the demographic model with the best fit was the exponential growth model (BF = 25.11).

Demographic history of the species (Fig 3B) depicts a population expansion that started at about 10 thousand years ago (ka), which coincided with a global climate warming corresponding to the first Marine Isotope Stage (MIS). Migrate-n analysis with the cyt b dataset resulted in no clear pattern of migration probably due to the small amount of variation among sequences, and thus it was not included in the paper.

### Microsatellite loci

Eight microsatellite loci (S1 Table) were analyzed for 155 of the 157 *R*. *nasutus* specimens collected in the Caatinga biome.

The number of alleles per locus ranged from 1 (R8) to 14 (List14-064). Since the R8 locus was monomorphic for all individuals, it was excluded from the analysis.

The basic statistics for the microsatellite data is summarized in Table 4. Three loci showed significant deviations from HWE in at least one population, possibly due to the admixture of null alleles or LD. Microsatellite loci analyses revealed moderate levels of genetic diversity and low allelic diversity. Null alleles were detected for loci List14-010 in Group 1 and List14-021 in Group 2. Significant LD between List-025 and List-064 loci, and List-025 and L43 were found in Group 1.

**Table 4:** Microsatellite genetic variation (seven loci) for the four groups of *R*. *nasutu*s.

	**Group 1 (ALT+CAM+PAR+PIR)**									
**Locus**	**R31**	**List14-010**	**List14-025**		**L43**	**List14-013**	**List14-021**	**List14-064**	**Average gene diversity**	***F***_**IS**_ **(*P* value)**
*N*	52	48	50		44	42	43	44	0.157± 0.153	-0.063 (0.851)
*N*_*A*_	1	2	1		5	6	4	10		
*P*_*A*_	0	0	0		0	5	2	3		
*H*_*O*_	ND	**0.234**	ND		**0.955**	0.381	**0.047**	0.795		
*H*_*E*_	ND	**0.491**	ND		**0.749**	0.372	**0.069**	0.728		
	**Group 2 (CAR+JAG)**									
*N*	68	68	68	68		64	68	67	0.255 ± 0.169	**0.122 (0.018)**
*N*_*A*_	2	3	2	4		2	2	6		
*P*_*A*_	1	1	0	0		1	0	1		
*H*_*O*_	0.015	0.044	0.162	0.485		0.266	**0.103**	0.328		
*H*_*E*_	0.015	0.044	0.152	0.543		0.255	**0.220**	0.326		
	**Group 3 (STA)**									
*N*	13	13	13	13		13	13	13	0.255 ± 0,169	-0.036(0.674)
*N*_*A*_	2	2	1	3		2	6	1		
*P*_*A*_	1	0	0	0		0	1	0		
*H*_*O*_	0.692	0.077	ND	0.462		**0.000**	0.615	ND		
*H*_*E*_	0.508	0.077	ND	0.394		**0.147**	0.658	ND		
	**Group 4 (SOU)**									
*N*	26	26	26	25		24	26	26	0.184 ± 0.136	-0.009(0,610)
*N*_A_	1	3	2	3		1	2	4		
*P*_A_	0	1	0	1		0	1	0		
*H*_*O*_	ND	0.077	0.115	0.640		ND	0.077	0.231		
*H*_*E*_	ND	0.147	0.111	0.525		ND	0.145	0.217		

*N*—sample size for each group; *N*_A_—number of alleles at each locus; *H*_O_ and *H*E—observed and expected heterozygosities, respectively; *P*_A_*—*private alleles; *F*_IS_—inbreeding coefficient; **ALT**–Altos, **CAM–**Campo Maior, **PAR–**Parnaíba, **PIR**–Piracuruca; **CAR**—Carnaúba dos Dantas; **JAG–**Jaguaruana; **SOU**–Sousa; **STA**—Serra Talhada. Significant deviations from Hardy–Weinberg equilibrium are shown in bold.

Among 155 individuals, 111 had unique genotypes: ALT+CAM– 22; PAR– 18; PIR– 8; CAR– 17; JAG– 25; STA– 9; SOU—15. There are two shared genotypes between CAR and JAG, one genotype shared between STA and JAG, one shared between PAR and PIR, and another shared between PAR and SOU. The highest number of shared genotypes was observed among *R*. *nasutus* specimens from Jaguaruana. In addition, positive *F*_IS_ values were significant in Group 2, which comprised Jaguaruana specimens.

**Fig 4:**
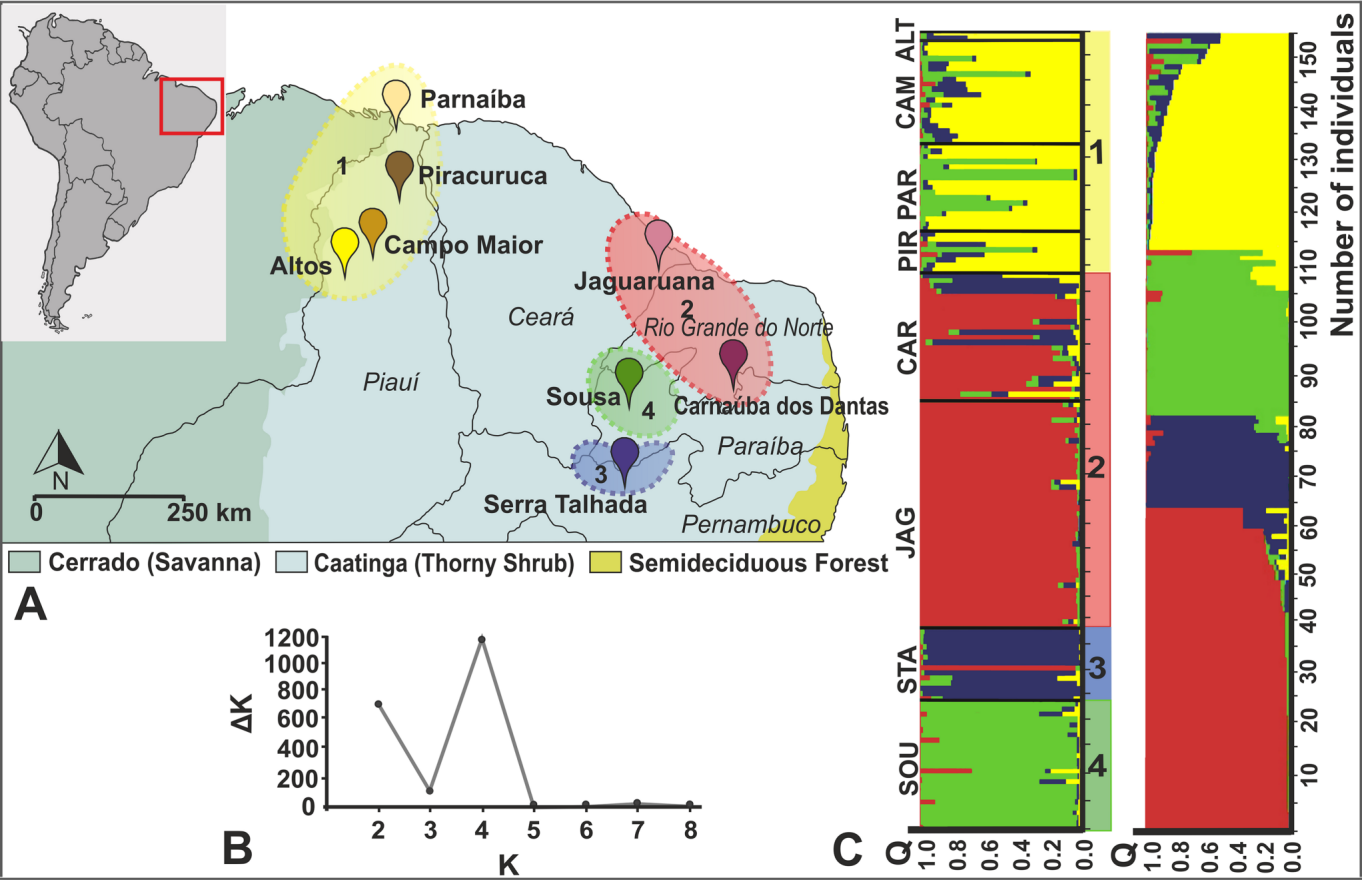
A—Sampling sites map of *R*. *nasutus* specimens; B– ΔK values are shown for K ranging between 2 and 8; C—population structure of *R*. *nasutus* estimated with Structure for *K* = 4 organized by geographic region (on the left) and by Q value (on the right). Each vertical bar represents the individual probability of a single bug to belong to a specific genetic cluster; the color(s) in each bar represent the proportion of the individual’s genome coming from each of the four assumed clusters. Black lines separate the sampling localities. Structure diagrams for *K* = 2–8 are presented as supporting information (S1 Fig).

The Bayesian clustering using seven microsatellite loci for 155 individuals revealed the existence of four groups (Fig 4). The ΔK method indicated 4 groups as the most likely population structure. Group 1 includes all sampling locations of *R*. *nasutus* in Altos (ALT), Campo Maior (CAM), Parnaíba (PAR), and Piracuruca (PIR). Group 2 is the most numerous and genetically variable and contains samples from Jaguaruana (JAG) and Carnaúba dos Dantas (CAR). Group 3 includes specimens from Serra Talhada (STA), and Group 4 is composed of specimens from Sousa (SOU). These groups were in accordance with the pairwise *F*_ST_ comparisons among sequences from different localities. Pairwise *F*_ST_ values between sampling localities (Table 3) revealed non-significant differences between Altos, Campo Maior, Piracuruca and Parnaíba, and also between Jaguaruana and Carnaúba dos Dantas. Pairwise *F*_ST_ values between Sousa, Serra Talhada and all other localities were significant.

The highest value of Shannon’s allele information index (^*S*^*H*_*A*_) describing genetic variation in population was shown in Group 1 (0.955). Group 1 also displayed the highest number of private alleles (10) over all loci. The other groups had a smaller number of private alleles: four private alleles were found in Groups 2 and 4, and only two private alleles were found in Group 3 (Table 4).

Genetic differentiation based on the *F*_ST_ and Shannon’s mutual information index ^S^*H*_UA_ between groups estimated in the Structure program [50] exhibited similar but not identical patterns (Table 5). The highest differentiation values were found between pairs of groups 2–4 (*F*_ST_ = 0.562, ^S^*H*_UA_ = 0.203), 3–4 (*F*_ST_ = 0.470, ^S^*H*_UA_ = 0.197) and 1–2 (*F*_ST_ = 0.438, ^S^*H*_UA_ = 0.270). The lowest values of *F*_ST_ were obtained for groups 1 and 3, although the lowest values of ^S^*H*_UA_ were indicated between groups 2 and 3. Also, relatively low values of *F*_ST_ and ^S^*H*_UA_ were found between groups 1 and 4 (0.277 and 0.144, respectively).

**Table 5:** ^S^*H*_UA_ indexes (above the diagonal), *F*_ST_ values (below the diagonal) and ^*S*^*H*_A_ indexes (in diagonal) among the four groups of *R*. *nasutus* determined based on seven microsatellite loci.

Pairwise *F*_ST,_^S^*H*_UA_	Group 1	Group 2	Group 3	Group 4
**Group 1**	0.955	0.270	0.159	0.144
**Group 2**	0.438*	0.584	0.122	0.203
**Group 3**	0.275	0.418*	0.624	0.197
**Group 4**	0.277*	0.562*	0.470*	0.431

Group 1: ALT—Altos (Piauí), CAM—Campo Maior (Piauí), PAR—Parnaíba (Piauí), PIR—Piracuruca (Piauí); Group 2: CAR—Carnaúba dos Dantas (Rio Grande do Norte), JAG—Jaguaruana (Ceará); Group 3: STA—Serra Talhada (Pernambuco), Group 4—SOU—Sousa (Paraíba).

* P < 0.05

Results of the Ewens-Watterson test indicated neutral evolution of all loci in all groups, but in Group 1, in which neutrality was rejected in locus L43 as observed homozygosity values were lower than expected (Table 6). Because past demographic events can cause significant departures from neutrality, we tested Group 1 for the occurrence of possible bottleneck events. Results of the analysis performed with the program Bottleneck did not indicate any signs of recent population decline among *R*. *nasutus* specimens from Group 1. One-tailed Wilcoxon sign-rank test for heterozygote excess indicated that each group was in mutation-drift equilibrium for all mutation models evaluated: IAM, TPM, and SMM (P > 0.05).

**Table 6:** Ewens–Watterson neutrality test.

	Group 1		Group 2		Group 3		Group 4	
Locus	*F*_*O*_	*F*_*E*_	*F*_*O*_	*F*_*E*_	*F*_*O*_	*F*_*E*_	*F*_*O*_	*F*_*E*_
R31	ND	ND	0.985	0.814	0.512	0.752	ND	ND
List14-010	0.515	0.810	0.510	0.690	0.926	0.748	0.388	0.456
List14-025	ND	ND	0.851	0.815	ND	ND	0.891	0.785
L43	**0.259**	**0.475**	0.461	0.582	0.621	0.584	0.486	0.631
List14-021	0.931	0.563	0.781	0.815	0.858	0.754	0.858	0.790
List14-064	0.255	0.266	0.688	0.448	0.367	0.318	0.786	0.444
List14-013	0.633	0.420	0.747	0.811	ND	ND	ND	ND

*F*_*O*_*−*observed homozygosity, *F*_*E*_*−*expected homozygosity. *F*_*O*_ and *F*_*E*_ values indicating significant difference (P < 0.05) are shown in bold.

**Fig 5:**
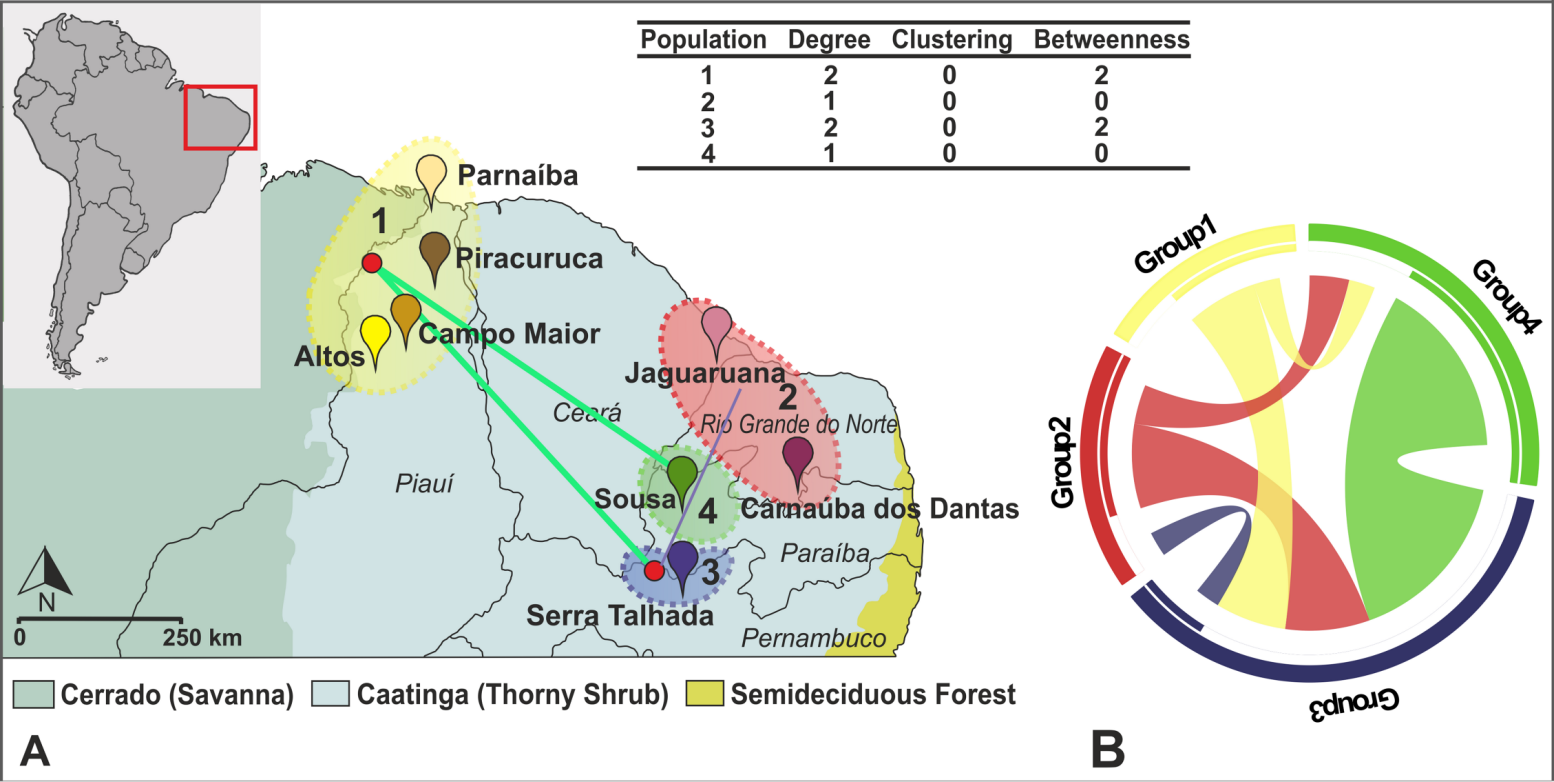
A—Populational network based on microsatellite data showing two populations (1 and 3) as source of migrants for all others, based on higher values of degree and betweenness components. Red circles represent the fundamental units of the system (i.e. source-sink populations). Each line represents the connection of a source population to a sink population. Line thickness is proportional to linkage strength of such relationships. B—Migration pattern of *R*. *nasutus* in the Caatinga region based on Migrate-n analysis. The color of each stripe in the middle of the circle represents the color of the source group of migrants. The width of each stripe is proportional to the number of migrants.

EDENetwork analysis revealed strong connections between Groups 1 and 3 and 1 and 4. Groups 2 and 3 were also connected. We did not observe any indication of substructuring within groups (Clustering = 0). Groups 1 and 3 were identified as possible source-sink populations based on the network analysis. These groups exhibited the highest connection values (calculated through the degree parameter) and betweenness, which indicate that they are exchanging migrants with other groups (Fig 5A).

Two of seven microsatellite loci (L43, List14-025) had complex repetition motifs that did not fit a SMM. Although List14-064 and List14-021 were reported with the same dinucleotide repeat motifs [43], we observed nucleotide substitutions (S2 Fig). Thus, we imposed the IAM mutation model as more appropriate for our data set for migration analysis. The estimates of the marginal likelihood (lmL) and the posterior model probability (*P*) for the 10 different models representing various scenarios of migration in Migrate-n revealed support for the 7^th^ model (Table 7). Migration pattern of *R*. *nasutus* in the Caatinga biome inferred by Migrate-n analysis with “full migration” model (Fig 5B) revealed the existence of bidirectional gene flow between Groups 2 and 3. Unidirectional gene flow was detected from Group 2 to 4, from Group 1 to 3, and from Group 4 to 3. Group 1 seems to be a source population, because it only provides emigrants to other Groups, whereas Group 3 is a sink population as it is composed by immigrants from the other three populations. AMOVA between east (Carnaúba dos Dantas, Jaguaruana, Sousa and Serra Talhada) and west (Altos, Campo Maior, Parnaíba and Piracuruca) populations shows that the percentage of within-population variation is higher (53.18%, Ф_ST_ = 0.46, P < 0.0001) than among populations within groups (33.51%, Ф_SC_ = 0.39, P < 0.0001) or between groups (13.31%, Ф_CT_ = 0.13, P < 0.21). The latter non-significant value of AMOVA statistic thus indicates gene flow between west and east groups.

**Table 7:** Model selection with Migrate-n.

No	Migration pattern	Harmonic lmL	*P*
1	1─>2─>3<─4<─1; 4<─>2	-487.39	0.00000
2	3─>1─>2<─3─>4	-709.12	0.00000
3	4<─>1─>3─>2; 1─>2	-571.86	0.00000
4	4<─>1<─>2; 1─>3	-632.87	0.00000
5	2<─>3<─4<─>2	-625.25	0.00000
6	1<─3<─>2─>4	-758.89	0.00000
**7**	**3<─1─>4─>3<─>2**	**-451.98**	**0.99999**
8	4<─1─>3─>2─>4─>3	-579.88	0.00000
9	1<─2─>3─>1; 4<─>2	-535.22	0.00000
10	4─>1<─2<─3─>1	-690.89	0.00000

Arrow heads indicate the direction of migration. The highest migration pattern probability is shown in bold. *No—*model number, *Harmonic lmL*—harmonic estimates of the marginal likelihood, and *P—*posterior probability for each model. Population numbers correspond to: 1 –Group 1; 2 –Group 2; 3 –Group 3; 4 –Group 4.

Linear regression analysis (S3 Fig) did not reveal a statistically significant correlation between cyt b-derived genetic distances and geographic distances (R^2^ = 0.05, P = 0.258). However, a weak correlation was observed between microsatellite-based *F*_ST_ distances and geographic distances (R^2^ = 0. 29, P = 0.003), suggesting that distance contributes ~29% to the shaping of genetic diversity [63].

## Discussion

This paper describes the population structure and demographic history of *R*. *nasutus* specimens collected from *C*. *prunifera* palm trees throughout most of the Caatinga biome. The pattern of genetic diversity found with the analyses of seven microsatellite loci and cytb sequences suggests that the colonization of the Caatinga by *R*. *nasutus* took place from west to east through the region (Fig 5), partially coinciding with a population expansion event that occurred during the last 12–10 thousand years (Fig 3).

### *R*. *nasutus* genetic diversity and population structure

The genetic structure revealed by microsatellite analyses (Figs 4 and 5) indicate the existence of four highly differentiated groups with pairwise *F*_ST_ values (>0.25; Table 5) well above those estimated for other triatomine populations at similar geographical scales, such as for *R*. *prolixus* in Venezuela [5] and *T*. *infestans* in Peru and Argentina [64, 65]. Although this could indicate low levels of gene flow between populations or even the possibility of cryptic speciation (as seen for other *Rhodnius* species [5]), results from the cyt b marker do not support the latter hypothesis. There is no evidence of present potential isolation by distance that could explain the population substructure observed herein. A weak correlation was found between genetic and geographical distances (S2 Fig), assuming that the correlation between geographic and genetic distances indicate isolation by distance (but see reference [66] for a discussion about the limitations of this analysis). Indeed, populations of Group 1 are geographically close and have low and non-significant pairwise *F*_ST_ values, but other populations such as Sousa and Carnaúba dos Dantas, which are also close, are highly structured (*F*_ST_ = 0.51, P < 0.01). Therefore, we can hypothesize that restricted gene flow in the Caatinga region might result from human modifications, since the main sylvatic ecotope of this species, *C*. *prunifera* palm trees, has been reduced significantly due to continuous deforestation [67]. Biodiversity losses in tropical areas have been strongly determined by deforestation, which can interrupt environmental patches that uphold connectivity with other habitats [68]. Future research considering the possible routes of human migration among the studied communities, as well as a more complete analysis of the distribution of *C*. *prunifera* palm trees are necessary to adequately address this hypothesis.

Reduced genetic diversity was detected in the three *R*. *nasutus* groups from eastern Caatinga (groups 2, 3 and 4) evidenced by the low number of alleles present (< 5) in the majority of the loci analyzed, and low heterozygosity levels. Reduced diversity commonly occurs in endemic species which have low effective population sizes caused by habitat fragmentation [69]. In this case, stochastic processes such as genetic drift and inbreeding within isolated patches are expected to shape the genetic background of populations [70]. Indeed, Group 2 shows moderate levels of inbreeding (*F*_IS_ = 0.122, P = 0.018).

Although two loci are monomorphic (List14-025 and R31), Group 1 from western Caatinga presents the highest heterozygosity levels. Moreover, this group has the highest Shannon’s allele information index ^*S*^*H*_*A*_ (0.955), further indicating its high variability. Two loci display heterozygote excess (L43 and List14-021), which might result from isolate-breaking (when two previously isolated populations are mixed [71]). It is important to note that Group 1 also has the highest number of private alleles. Perhaps the high number of private alleles observed in this group results from long-term isolation during glaciation periods.

Evidence for departures from neutrality and bottleneck events were not detected in the *R*. *nasutus* populations analyzed. However, this result should be interpreted with caution, since the small sample size of some locations could prevent proper testing of this hypothesis [72]. Moreover, the reduction of effective population size, heterozygosity levels and allelic diversity may go undetected when (i) they occurred for a limited time interval or very recently [73], (ii) are followed by a sudden population growth [74], or (iii) when populations experienced low levels of subsequent immigration [75].

### Gene flow among groups and colonization scenarios based on microsatellite data

A scattered mosaic of habitat fragments, such as observed in the Caatinga region, might cause population reduction due to an impact in the connectivity of habitat networks, favoring dispersal through a series of successive stepping-stones events [76, 77]. Network and Bayesian coalescent analyses indicated a western-eastern colonization of the biome. Group 1 is a source population, from which individuals emigrated to Groups 3 and 4, while Group 3 is a sink population, also receiving emigrants from Groups 2 and 4 (Fig 5A). Geographically close groups of eastern Caatinga have a complex pattern of gene exchange, with unidirectional migration of Group 2 individuals to Group 4, and Group 4 individuals to Group 3, as well as bidirectional gene flow between Groups 2 and 3 (Fig 5B).

Unidirectional gene flow may be explained by a decrease in capacity at the source habitat and availability of expansion potential at the target area [78]. The niche capacity of *R*. *nasutus* may be limited by several ecological factors, such as the sparse distribution of preferred food sources (fauna associated with the palms) and human activity.

### Phylogeography, demographic history based on mitochondrial DNA sequence analysis

The network shows that most haplotypes differ by a single substitution, indicating reduced diversity. These haplotypes seem to be under neutral evolution, since 13 out of 16 variable sites resulted in synonymous substitutions. Two haplotypes are shared by five geographically distant locations (Serra Talhada, Parnaíba, Jaguaruana, Campo Maior and Altos), which could indicate high gene flow among populations or reflect retention of ancestral polymorphism [79]. The remaining 14 haplotypes are exclusive (Fig 2), and thus support restricted migration. Pairwise *F*_ST_ comparisons corroborate the latter hypothesis, as 24 out of 28 values are high (> 0.6) and significant (Table 3).

Populations can become structured quickly during a range expansion event as a consequence of the colonization of newly available patchy areas by pioneering groups with reduced population sizes [76]. In the case of a patchy environment, there are limitations to dispersal and therefore the colonization of new habitats will increase genetic structure among populations as only a subset of the original gene pool will be transferred [72].

Evidence suggests that populations of *R*. *nasutus* expanded from west to east throughout the Caatinga in the last 12–10 ka. Molecular diversity indices showed high haplotype diversity (Hd) and low nucleotide diversity (π), which indicates that despite the high number of different haplotypes per population, they only differ by 1–5 nucleotides. This observation is consistent with expansion of source populations with small effective size [79]. Mismatch distribution analysis showed a good fit to the expected under the model of population growth (Fig 3A). Moreover, the demographic model that better represents the population dynamics of *R*. *nasutus* is the exponential growth (BF = 25.11), evidenced by a 5-fold increase in the species population size in the Holocene (Fig 3B).

The demographic history of *R*. *nasutus* is estimated to have begun 66 thousand years ago thus covering Pleistocene–Holocene geological epochs (Fig 3B). Based on the Bayesian Skyline Plot results, the population size of *R*. *nasutus* was stable for approximately 50,000 years. This demographic stability may reflect the presence of a putative refuge area for Seasonally Dry Tropical Forests in the Caatinga, spanning most of the species current distribution area during the Last Glacial Maximum (24–12 ka) [16].

*R*. *nasutus* went through a population expansion 12–10 ka, during the Holocene. This expansion is consistent with global climate changes (Fig 3B), as it coincides with the end of the largest dry season in South America, during the glaciation Wisconsin-Würm (18–12 ka). This period is characterized by the expansion of semi-humid vegetation in South America and diversification of Neotropical biota in seasonally dry areas [80, 81]. Moreover, the Caatinga biome experienced an increase in moisture levels during the late Pleistocene (12–6 ka), which possibly contributed to the expansion of *Mauritia* palm tree species in the region [82] and likely also *Copernicia* palms.

### Incongruence between nuclear and mitochondrial data

The genetic structure of *R*. *nasutus* populations inferred based on the two markers differed considerably (Figs 2 and 4). Cyt b analysis indicated more complex substructure than the microsatellite data (Table 3). Although Group 3 (Serra Talhada) and 4 (Sousa) remained separate by both microsatellites and cyt b data, microsatellite-defined Group 1 comprises four populations (Altos, Campo Maior, Piracuruca and Parnaíba), and Group 2 includes two populations (Jaguaruana and Carnaúba dos Dantas), whereas cyt b data grouped together Altos and Campo Maior with the geographically distant population of Jaguaruana. Assuming that sampled populations represent panmitic units, we might speculate that in the past, Altos, Campo Maior and Jaguaruana were connected, while Piracuruca and Parnaíba were separated. In a contemporary event, the Jaguaruana population became isolated from the western group, which clustered with the other two western populations, Piracuruca and Parnaíba. It is worth mentioning, however, that *F*_ST_ results based on cyt b data should be interpreted with caution, due to the low divergence found between *R*. *nasutus* sequences. In a contemporary event, the Jaguaruana population became isolated from the western group, which clustered with the other two western populations, Piracuruca and Parnaíba. Group 1 heterozygote excess and pairwise cyt b and microsatellite *F*_ST_ values among populations (0.20–0.77 and 0.07–0.24, respectively), thus corroborate the hypothesis of an isolate-breaking effect.

Incongruent results may also be explained by different evolutionary mechanisms (inheritance modes, population size, rates of evolution, response to selective pressure) and intrinsic biological peculiarities of triatomines (e.g. males fly more than females). Poor accordance between population genetic structures inferred from mitochondrial and microsatellite markers have also been reported for various animal groups, including mammals [83], amphibians [84] and insects [85].

### Dispersal capacity and epidemiological implications

*R*. *nasutus* was able to recently disperse within the Caatinga in response to favorable climatic events and environmental conditions. The species is presently facing an important ecological impact caused by the continuous deforestation of *C*. *prunifera* palm trees. Perhaps this ecological disturbance is contributing to the observation that *R*. *nasutus* now colonizes other species of palms (*A*. *speciosa*, *M*. *flexuosa*, *S*. *oleracea*, *A*. *intumescens* [12, 13]) and occasionally, even trees (*L*. *rigida* [14]). These observations show that *R*. *nasutus* populations have the ability to adapt to new microhabitats. In addition, it also invades human-modified areas and sporadically colonizes dwellings [10, 11, 86]. We observed that *C*. *prunifera* palm trees in deforested areas seem to be more prone to become infested with *R*. *nasutus* than those in forested areas (87% and 58%, respectively), which raises the risk of *T*. *cruzi* transmission to humans.

Demographic history data indicates the capacity of *R*. *nasutus* to increase its population size when environmental conditions are favorable. Therefore, closer attention should be paid to the distribution of *R*. *nasutus*, as it can maintain *T*. *cruzi* transmission within the Caatinga. Given the limitations inherent to insecticide spraying such as continuous re-infestation of insecticide-treated households by abundant native vectors, molecular studies focusing on the distribution patterns and demographic trends of sylvatic triatomines should be conducted in order to further improve the effectiveness of Chagas disease control.

## Supporting information

S1 Table Microsatellite loci used in this study. (DOCX)

S2 Table Molecular divergence of *R*. *nasutus* cyt b sequences from the same locality (in bold) and from different localities. N–number of sequences analyzed.
(DOCX)

S1 Fig Population structure of *R*. *nasutus* estimated with Structure for *K* = 2–8. (TIF)

S2 Fig Electropherograms of four different alleles of *locus* List14-064. (JPG)

S3 Fig Linear regression analysis between logarithms of pairwise *F*_ST_/(1 –*F*_ST_) ratios and geographic distances. (TIF)
