# Supplementary material for: Phylogeography and demographic history of the Chagas disease vector Rhodnius nasutus (Hemiptera: Reduviidae) in the Brazilian Caatinga biome
Source: PLoS Negl Trop Dis. 2018 Sep 24;12(9):e0006731. doi: 10.1371/journal.pntd.0006731 (PMC6195287; doi:10.1371/journal.pntd.0006731)
Supplement: S1 Table — (DOCX) [file pntd.0006731.s001.docx]

**S1 Table.** Microsatellite loci used in this study.

| **Locus** | **Sequence** | **Repeat** | **Range** | **Ta** | **Dye** | **Reference** |
| --- | --- | --- | --- | --- | --- | --- |
| **R8** | F: ATGGCAACTTTAATTTCAAGTATTC  R: TCTGACGAAACGCCACTG | (GT)_8_ | 131-133 | 50 | HEX | [44] |
| **R31** | F: TGTGGTAAGTCCTGTGTAGAAGG  R: TCTGTTGGTCCAGACACGG | (GT)_11_ | 203-205 | 52 | FAM | [44] |
| **L43** | F: ACAGGTTGTACAGCGCGTC  R: CATGTTCCGTCACGTAGGC | (GT)_3_N_8_(CT)_2_(GT)_13_ | 114-135 | 58 | HEX | [45] |
| **List14-010** | F: AATGATGACTGTATTGATGGGC  R: TTCGACCAACAACAACTTCCC | (CA)_9_ | 311-339 | 52 | FAM | [46] |
| **List14-013** | F: CATACTACACGCACACAAGACC  R: ATACTCGCATCAAGCCATTTGG | (AC)_10_ | 335-345 | 55 | FAM | [46] |
| **List14-021** | F: AACCTCTGAACACATCAAATGG  R: AGCTACCTCTTGCCTCTACG | (GT)_8_ | 291-299 | 55 | HEX | [46] |
| **List14-025** | F: CCGCTCTATCAACTACTCC  R: GATCCCTTATGTTTCTCAGC | (TC)_9_(AC)_7_N_13_(AC)_7_ | 163-181 | 50 | HEX | [46] |
| **List14-064** | F: AGAAAATGAGCAAAACGGCC  R: ACAGGCAAACAACTATGACG | (GT)_10_ | 237-247 | 57 | HEX | [46] |

*Ta* annealing temperature
