# Supplementary material for: Phylogeography and demographic history of the Chagas disease vector Rhodnius nasutus (Hemiptera: Reduviidae) in the Brazilian Caatinga biome
Source: PLoS Negl Trop Dis. 2018 Sep 24;12(9):e0006731. doi: 10.1371/journal.pntd.0006731 (PMC6195287; doi:10.1371/journal.pntd.0006731)
Supplement: S2 Table — N–number of sequences analyzed. (DOCX) [file pntd.0006731.s002.docx]

**S2 Table**. Molecular divergence of *R. nasutus* cyt b sequences from the same locality (in bold) and from different localities. N – number of sequences analyzed.

|  | 1 | 2 | 3 | 4 | 5 | 6 | 7 | 8 |
| --- | --- | --- | --- | --- | --- | --- | --- | --- |
| 1. ALT (N = 4) | **0.001 (± 0.001)** |  |  |  |  |  |  |  |
| 2. CAM (N = 16) | 0.001 (± 0.001) | **0.001 (± 0.001)** |  |  |  |  |  |  |
| 3. CAR (N = 25) | 0.003 (± 0.002) | 0.002 (± 0.002) | **0.000** |  |  |  |  |  |
| 4. JAG (N = 32) | 0.001 (± 0.001) | 0.001 (± 0.000) | 0.002 (± 0.002) | **0.000** |  |  |  |  |
| 5. PAR (N = 18) | 0.007 (± 0.003) | 0.006 (± 0.003) | 0.008 (± 0.003) | 0.006 (± 0.003) | **0.002 (± 0.001)** |  |  |  |
| 6. PIR (N = 9) | 0.005 (± 0.002) | 0.004 (± 0.002) | 0.006 (± 0.003) | 0.004 (± 0.002) | 0.004 (± 0.002) | **0.002 (± 0.001)** |  |  |
| 7. SOU (N = 28) | 0.003 (± 0.002) | 0.002 (± 0.002) | 0.003 (± 0.002) | 0.002 (± 0.002) | 0.008 (± 0.003) | 0.006 (± 0.003) | **0.000** |  |
| 8. STA (N = 15) | 0.003 (± 0.002) | 0.003 (± 0.002) | 0.004 (± 0.003) | 0.003 (± 0.002) | 0.005 (± 0.002) | 0.003 (± 0.002) | 0.004 (± 0.002) | **0.001 (± 0.001)** |
