# Supplementary figures and images for: Phylogeography and demographic history of the Chagas disease vector Rhodnius nasutus (Hemiptera: Reduviidae) in the Brazilian Caatinga biome
Source: PLoS Negl Trop Dis. 2018 Sep 24;12(9):e0006731. doi: 10.1371/journal.pntd.0006731 (PMC6195287; doi:10.1371/journal.pntd.0006731)

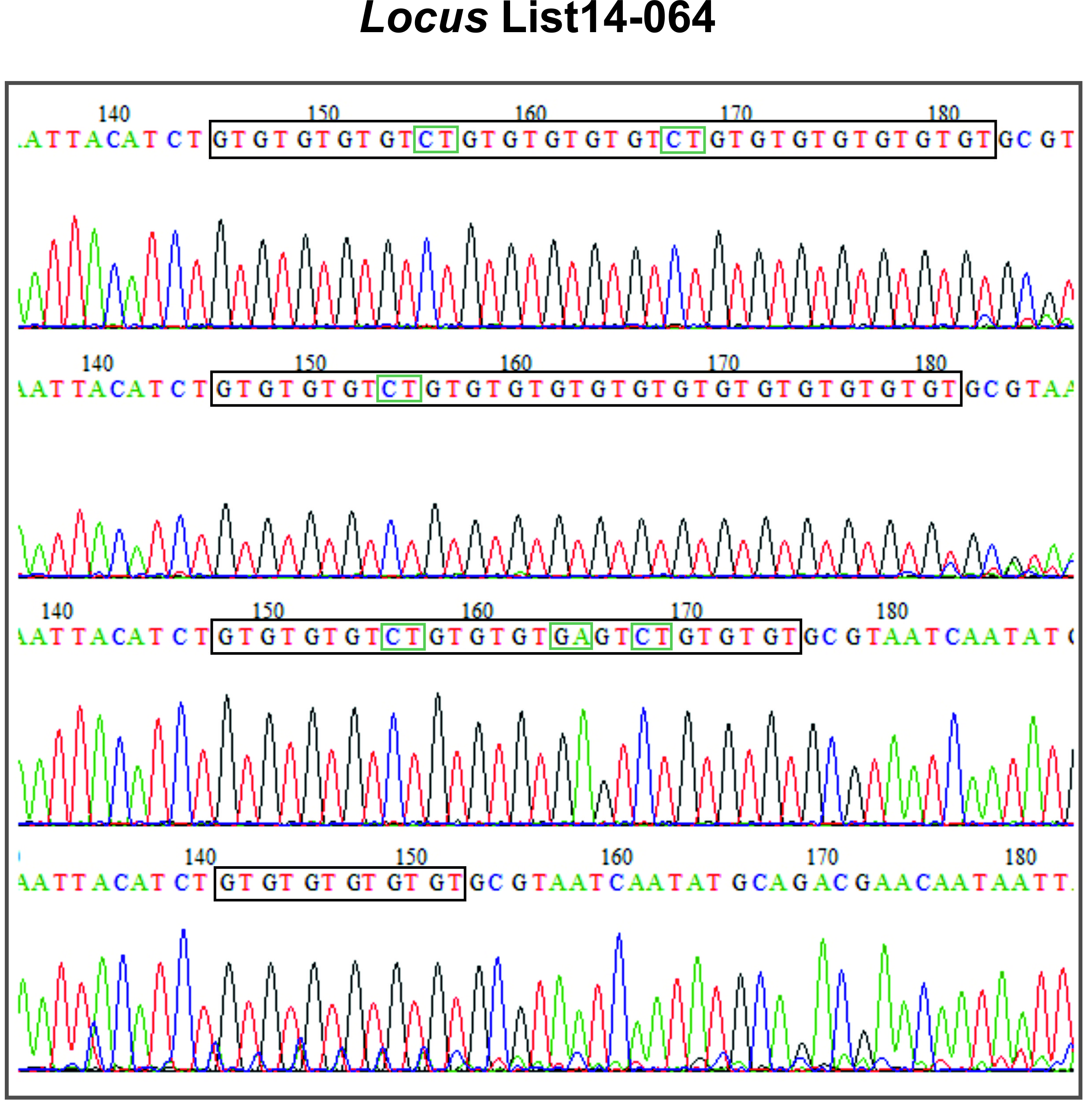

Supplement: S2 Fig — (JPG) [file pntd.0006731.s004.jpg]
